# Risk factors associated with HTLV-1 vertical transmission in Brazil: longer breastfeeding, higher maternal proviral load and previous HTLV-1-infected offspring

**DOI:** 10.1038/s41598-018-25939-y

**Published:** 2018-05-17

**Authors:** Arthur M. Paiva, Tatiane Assone, Michel E. J. Haziot, Jerusa Smid, Luiz Augusto M. Fonseca, Olinda do Carmo Luiz, Augusto Cesar Penalva de Oliveira, Jorge Casseb

**Affiliations:** 1 Instituto de Medicina Tropical de São Paulo, Universidade de São Paulo, Lab. de Imunologia e Dermatologia, LIM-56 SP, Brazil; 2 Hospital Universitário “Prof. Alberto Antunes”, Universidade Federal de Alagoas, Alagoas, Brazil; 3 Instituto de Infectologia “Emilio Ribas”, Secretaria da Saúde do Estado de São Paulo, São Paulo, SP Brazil; 4 Faculdade de Medicina FMUSP, Serviço de Alergia e Imunologia, Universidade de São Paulo, SP, Brazil; 5 Faculdade de Medicina FMUSP, Departamento de Medicina Preventiva, Universidade de São Paulo, SP, Brazil; 6 Department of Preventive Medicine, University of São Paulo Medical School, São Paulo, Brazil

## Abstract

HTLV-1 is transmitted primarily either through sexual intercourse or from mother to child. The mother/child pairs were classified as seroconcordant or serodiscordant. We analyzed mother to child transmission (MTCT) according to sociodemographic, clinical and epidemiological characteristics of the mother, child’s gender and duration of breastfeeding. Between June 2006 and August 2016 we followed 192 mothers with HTLV-1 infection (mean age 41 years old), resulting in 499 exposed offspring, 288 (57.7%) of whom were tested for HTLV-1, making up the final sample for the study, along with their 134 respective mothers. Among the tested mother/child pairs, 41 (14.2%) were HTLV-1 positive, highlighted that seven of 134 family clusters concentrated 48.8% of positive cases. Variables associated with a positive child: breastfeeding duration ≥12 months, maternal PVL ≥100 copies/10^4^ PBMC, mother’s age at delivery >26 years old, and HTLV-1 in more than one child of the same mother. In a multiple logistic regression, breastfeeding ≥12 months, higher maternal PVL and ≥2 previous HTLV-1-infected children remained independently associated with the outcome. Thus, high maternal PVL and breastfeeding beyond 12 months were independently associated with MTCT of the HTLV-1 infection. Our results reinforce the need for both prenatal HTLV screening in endemic areas and for advising mothers on breastfeeding.

## Introduction

Human T-cell lymphotropic virus type 1 (HTLV-1), a virus infecting humans since ancient times, is the causative agent of a lymphoproliferative malignancy named Adult T-cell Leukemia/Lymphoma (ATLL), as well as of the HTLV-1-associated myelopathy/tropical spastic paraparesis (HAM/TSP); HTLV-1 has also been associated with uveitis, infective dermatitis, and other inflammatory disorders^1–4^. This infection is endemic in many parts of the world, including southwestern Japan, South America, some of the Caribbean islands, western and central Africa, Australo-Melanesia and some areas of Middle-East, such as regions of Iran^5^. There are at least an estimated five to 10 million HTLV-1 carriers worldwide^3^, but screening of the population for the presence of the virus is performed for only one third of the endemic areas. Brazil is most likely the country with the highest absolute number of seropositive individuals, with almost one million infected people^6^.

HTLV-1 transmission primarily occurs through the following routes: 1) vertically (from mother to child), including a long time of breastfeeding (8); 2) sexual contact, primarily from men to women; and 3) parenterally through the transfusion of contaminated blood and blood components or through contaminated needles^7^. Concerning mother-to-child-transmission (MTCT) transmission, a review^8^ pointed out that in infants breastfed, the MTCT occurs at rates varying from 7.4%^9^ to 32%^10^, compared with a rate of less than 5% among bottle-fed children, some of them tested during adulthood^9,11–14^. Although prolonged breastfeeding seems to play a central role in the spread and maintenance of endemicity^9,12,14^, the relative importance of HTLV-1 main routes of transmission in Brazil is still a matter of discussion^15,16^. Preventive measures, such as prenatal screening and abstinence from breastfeeding by carrier mothers might reduce the prevalence of HTLV carriers, such as in Nagasaki population, in which a decline from 20–25% to 4% in the prevalence of HTLV carriers was observed. In Brazil, unfortunately, prenatal screening for HTLV has not yet been implemented throughout the country. In addition, there are no specific studies establishing the rate of MTCT transmission of the virus in the country, or the role of the HTLV-1 maternal proviral load as a risk factor for mother-to-child transmission. Analyses accounting simultaneously for possible epidemiological, clinical and biomolecular markers for vertical transmission are thus needed to assess any independent effect and may increase our understanding of the determinants of MTCT.

## Material and Methods

### Study Design and Study Population

A cross-sectional study involving mothers with HTLV-1 infection at the Institute of Infectology “Emílio Ribas” in São Paulo from June 2006 to August 2016 and their exposed child tested for HTLV infection. Mothers were excluded if they did not have any child tested or if their documented seroconversion occurred after the birth of their children. We classified the mother-child pairs into two groups: a) seropositive mother and her seropositive child tested and b) seropositive mother with her seronegative child tested.

### Diagnostic algorithm

The serological tests were carried out in the clinical laboratory at the Instituto de Infectologia Emilio Ribas, at its immunology section, employing the GOLD ELISA HTLV-1/2 (Immunoenzyme Test) and Western Blot Test (WB) (MP Diagnostics (MPD) HTLV Blot 2.4), for HTLV screening and confirmation, respectively. All WB positive cases were also submitted to a qualitative polymerase chain reaction (Nested-PCR) test^17^.

### HTLV-1 proviral load (PVL)

The HTLV-1 proviral load was quantified by real-time PCR, using primers and probes targeting the *pol* gene: SK110 (5′-CCCTACAATCCAACCAGCTCAG-3′, HTLV-1 nucleotide 4758–4779 (GenBank accession No. J02029), and SK111 (5′-GTGGTGAAGCTGCCATCGGGTTTT-3′, HTLV-1 nucleotide 4943–4920). The internal HTLV-1 TaqMan probe (5′-CTTTACTGACAAACCCGACCTACCCATGGA-3′) was selected using the Oligo (version 4, National Biosciences, Plymouth, MI, USA) and Primer Express (Perkin-Elmer Applied Biosystems, Boston, MA, USA) software programs and checked through a search of GenBank. The probe was located between positions 4829 and 4858 of the HTLV-1 genome and carried a 5′ reporter dye FAM (6-carboxy fluorescein) and a 3′ quencher dye TAMRA (6-carboxy tetramethylrhodamine). For quantification of the human albumin gene, the primers Alb-S (5′-GCTGTCATCTCTTGTGGGCTGT-3′) and Alb-AS (5′-AAACTCATGGGAGCTGCTGGTT-3′) and the albumin TaqMan probe (5′-FAMCCTGTCATGCCCACACAAATCTCTCCTAMRA-3′) were used as described previously^18,19^. Albumin DNA was quantified jointly in all samples in order to determine the amount of DNA used as an endogenous reference to normalize variations due to differences in PBMC counts or DNA extraction. The 25-μl PCR mixture for HTLV-1 or albumin DNA amplifications consisted of 5 μl DNA extract, primers SK110 and SK11 or Alb-S and Alb-AS (10 nM of each), 10 nM HTLV-1 or albumin TaqMan probe, TaqMan® Universal Master Mix II (Applied Biosystems®, Foster City, CA). For both the HTLV-1 and albumin DNA amplifications, after one cycle at 50 °C for 2 min and one cycle at 95 °C for 10 min, a two-step PAC procedure was used consisting in 15 s at 95 °C and 1 min at 60 °C for 45 cycles. Amplifications were carried out using the ABI 7300Fast Real-Time PCR System (Applied Biosystems®, Foster City, CA). The HTLV-1 copy number in each clinical sample was estimated by interpolation from the plasmid regression curve. To determine the proviral load, the HTLV-1 DNA copy number was normalized to the amount of the cellular albumin of the clinical sample, which was quantified in parallel. All samples were run in duplicates. Results were expressed as HTLV-1 DNA copies/10^4^ PBMCs, as described elsewhere^13^. Based on the median of asymptomatic individuals, 200 copies/10^4^ PBMCs of PVL was the value used as a cut off to discriminate from HAM/TSP subjects^20^.

### Statistical analysis

The categorical variables included in the univariate analysis were “having HTLV-1 infected mother or sibling”, HAM/TSP, ATL, race/ethnicity, place of birth, hepatitis C virus infection (HCV) status, history of blood transfusion and educational level. We transformed numerical variables into categorical variables for the analysis of mother-to-child transmission, with their mean or median serving as reference; for PVL, ≥100 copies/10^4^ PBMC were considered high; for breastfeeding duration, which averaged 11 months, the cut-off point adopted was 12 months.

Statistical analysis was conducted using Student’s t-test for parametric data, and the chi-square test for proportions. Possible differences in patient characteristics or laboratory values among the groups were evaluated with two-way Mann-Whitney’s test. Bivariate logistic analysis was performed to identify independent variables associated with HAM/TSP. Variables associated with the outcome at a significance level of p < 0.20 (HAM/TSP) in the bivariate analysis were included in a multiple logistic regression model; the only exception to this procedure was the inclusion of the variable gender, which was included regardless of its statistical significance in the bivariate model. Logistic analysis was performed with the aid of Stata 10 software (StataCorp. 2009. *Stata: Release 10*. Statistical Software. College Station, TX). We extracted all analyzed variables from our REDCap database (Valderbilt University, US) and used the statistical software Stata/IC 13.1 for the statistical analysis. *P* values < 0.05 were considered statistically significant. Variables reaching a significance level of *p* < 0.20 in the univariate analysis were included in a multiple logistic regression analysis, which we used to determine those that were independently and significantly associated with the outcome.

### Ethics

The Research Ethics Committee of the Faculty of Medicine of the University of São Paulo approved the current study under the number 407/12. All patients signed an informed consent form. The performance of tests was conducted according with their norms and regulations.

## Results

Among 192 mothers with HTLV-1 infection and their 499 respective exposed children, only 288 (57.7%) attended our invitation and were tested for HTLV-1, 41 of whom were positive for the virus, resulting in a transmission rate of 14.2%. Mothers’ age at delivery ranged from 14 to 48 years, with an average of 26.1 (±6.4) years. Maternal PVL ranged from 0 to 1221 copies/10^4^ PBMC, with a mean of 128 copies/10^4^ PBMC (±262); the number of children by mother ranged from 1 to 10, with a mean of 3.4 (±1.5) (Table 1).

**Table 1:** Prevalence of risk factors for HTLV-1 seropositivity among 288 subjects exposed to mother-to-child transmission.

Variables	Total		HTLV-1 seropositive child				
	N	%	N	%	OR	IC_95%_	*p*
Gender							
female	150	52.08	21	14.00	1		
male	138	47.92	20	14.49	1.04	0.54–2.02	0.905
Duration of breastfeeding							
<12 months	145	50.35	7	4.83	1		
≥12 months	143	49.65	34	23.78	6.15	2.62–14.41	**<0**.**001**
Sibling with HTLV-1							
no	247	85.76	13	5.26	1		
yes	41	14.24	20	48.78	17.14	7.48–39.27	**<0**.**001**
Mother’s proviral load							
<100 copies/10^4^ PBMC	196	68.06	21	10.71	1		
≥100 copies/10^4^ PBMC	92	31.94	20	21.74	2.31	1.18–4.53	**0**.**014**
Delivery							
vaginal	121	42.01	22	18.18	1		
cesarean	74	25.69	7	9.46	0.47	0.19–1.16	0.102
Number of children							
≤3 children	174	60.42	21	12.07	1		
>3 children	114	39.58	20	17.54	1.55	0.79–3.01	0.196
Mother with HAM/TSP							
no	173	60.28	27	15.60	1		
yes	114	39.72	14	12.28	0.76	0.38–1.51	0.432
Mother has mother/sibling HTLV-1+							
no	271	94.10	35	12.91	1		
yes	17	5.90	6	35.29	3.68	1.28–10.57	**0**.**016**
Husband HTLV-1+							
no	95	33.33	9	9.47	1		
yes	72	25.26	12	16.67	1.91	0.76–4.82	0.170
No husband or not tested	118	41.40	19	16.10	1.83	0.79–4.26	0.159
Blood transfusion history							
No	266	92.36	40	1504	1		
yes	22	7.64	1	18.18	0.27	0.35–2.06	0.206
Maternal co-infections							
none	256	88.89	38	14.84	1		
HCV	18	6.25	1	5.55	0.34	0.04–2.61	0.98
HIV-1	9	3.12	2	22.22	1.64	0.33–8.19	0.547
Other	5	1,74	0				

From 288 tested children, 253 had been breastfed, and the duration of breastfeeding ranged from less than two weeks to 60 months (mean 10.7 months). Co-infections were detected in 19 mothers. We identified a tendency to familial aggregation of cases, where among 134 tested mother/child pairs, 41 (14.2%) were HTLV-1 positive, highlighted that seven of 134 family clusters concentrated 48.8% of positive cases. Table 2 present the prevalence and *Odds Ratio* (OR) for HTLV-1 seropositivity among 288 children exposed to vertical transmission according to the child’s gender, duration of breastfeeding, and maternal clinical and epidemiological characteristics.

**Table 2:** Demographical characteristics for HTLV-1 seropositivity among 288 subjects exposed to mother-to-child transmission.

Variables	Total		HTLV-1 seropositive child				
	N	%	N	%	OR	IC_95%_	*p*
Mother’s age at delivery							
≤26 years	137	47.57	11	8.03	1		
>26 years	151	52.43	30	19.87	2.84	1.32–5.92	**0**.**005**
Ethnicity/Race							
white	152	52.78	23	15.13	1		
mestizo	87	30.21	8	9.19	0.57	0.24–1.33	0.193
black	38	13.19	6	15.79	1.05	0.39–2.79	0.920
asian	8	2.78	4	50.00	5.61	1.31–24.03	**0**.**020**
amerindian	3	1.04	0				
Marital status							
married	161	52.90	25	15.53	1		
single	18	6.25	1	5.55	0.32	0.40–2.51	0.279
widower	57	19.79	5	8.77	0.52	0.19–1.44	0.209
separated	31	10.76	2	6.45	0.37	0.08–1.67	0.199
divorced	21	7.29	8	38.09	3.35	1.26–8.91	**0**.**016**
Mother’s birth place (Region)							
Midwest	12	4.17	2	16.67	1		
Northeast	96	33.33	16	16.67	1	0.20–5.00	1.000
Southeast	168	58.33	23	13.69	0.79	0.16–3.85	0.774
South	10	3.47	0				
North	2	0.70	0				
Education							
elementary (incomplete)	125	45.96	19	15.20	1		
elementary (complete)	62	22.79	10	16.13	1.07	0.47–2.47	0.869
high school (incomplete)	9	3.31	1	11.11	0.70	0.82–5.90	0.741
high school (complete)	56	20.59	5	8.93	0.55	0.19–1.54	0.256
college	20	7.35	1	5.00	0.29	0.04–2.33	0.246

Variables associated with an HTLV-1-seropositive offspring were: breastfeeding for 12 months or more (*p* < 0.001), having two or more HTLV-1 positive children(*p* < 0.001), mother’s PVL (≥100 copies/10^4^ PBMC) (*p* = 0.014), mother with an epidemiological history suggestive of virus acquisition by mother-to-child transmission (her mother and/or sibling with HTLV-1 infection) (*p* = 0.016), age (*p* = 0.005), Asian maternal race (*p* = 0.02) and divorced marital status (*p* = 0.016). The odds ratio of possible protection conferred by cesarean delivery did not reach statistical significance (Table 1). Likewise, the mother’s schooling was inversely proportional to the child’s risk of seropositivity, but this association was not statistically significant (Table 2). Variables associated with the outcome at a significance level of p < 0.20 (MTCT) in the univariate analysis were included in a multivariate logistic model. On that model, variables independently associated with child seropositivity were: maternal proviral load ≥100 copies/10^4^ PBMC (*p* = 0.005), breastfeeding for 12 months or more (*p* < 0.001), and having two or more HTLV-1-infected children (*p* < 0.001) (Table 3).

**Table 3:** Multiple logistic regression analysis to identify HTLV-1 seropositive predictors among vertically exposed children.

Variables	OR	IC_95%_	*P*
Maternal proviral load	3.26	1.43–7.44	0.005
Duration of breastfeeding	6.66	2.54–17.46	<0.001
Sibling with HTLV-1 infection	17.63	6.97–44.59	<0.001

## Discussion

For the first time in Brazil, in this study we simultaneously analyzed epidemiological, clinical markers, and maternal proviral load, for mother-to-child HTLV-1 transmission. The prevalence of concordant transmisson to the child was 14.2%, which was lower than found in countries such as Jamaica (18% to 22%)^21,22^, Peru (18%)^23^, Iran (16.6%)^24^, Gabon (17.5%)^25^, Martinique (27%)^26^, and Japan between 1986 to 1991^27^ before implementing screening policies among pregnant women. In contrast, it was higher than in French Guiana (9.7%)^28^, and in Japan by the end of the 1990s (3.9%)^27^. This variability of transmission rates to the offspring between different populations, and the lower rates in countries that have implemented control measures, strongly suggest the predominant role of variables related to breastfeeding in HTLV-1 transmission^27,28^.

We addressed several risk factors for mother/child positive pairs, including maternal load of circulating HTLV-1. The risk of this kind of transmission increases exponentially in the presence of higher PVLs^21^, and in the present study women with PVL >100 copies/10^4^ PBMC presented a higher transmission risk.

In agreement with our results a similar study done in Jamaica showed an association between prolonged breastfeeding and MTCT. Breastfeeding over 12 months was also associated with a risk of 32% transmission compared with only 9% for a shorter duration of breastfeeding^10^. In those children, the median time of HTLV-1 infection was estimated at 12 months^29^. Among children of Peruvian women with HTLV-1 the risk was 15.1 times greater for those who were breastfed during 12 to 24 months compared to less than six months^30^. One possible explanation would be that passive transfer of maternal antibodies during pregnancy may inhibit HTLV-1. However, those antibodies decline and disappear six to 12 months after birth^31^.

Overall, the mother/child positivity rate was 14.2%, reaching 50% for infected Asian-descendant mothers. However, the positive association between Asian ethnicity and seropositive child on the univariate analysis was not confirmed on the multivariate analysis. This higher transmission probably occurred because of the longer breastfeeding time in this group (24–36 months). The only one of the six positive children who had been breastfed for less than 24 months (six months) had a mother with a proviral load of 488 copies/10^4^ PBMC. In fact, both the high mother PVL and prolonged breastfeeding were independently associated with transmission to the offspring. Divorced marital status was another possible confounding factor, which also did not remain in the multivariate analysis. Unfortunately, the small sample of this specific subgroup is not strong evidence and deserves further investigation of divorce as a risk for vertical transmission.

Mother’s age (>26 years) at the time of delivery was associated with seropositivity in the child, and one of the explanations could be the increase in the prevalence of HTLV-1 infection with increasing age, observed mainly among women^32^, for whom sexual transmission is more efficient^33^. This can also be due to the time between the beginning of the relationship with an infected partner and seroconversion. The seropositivity of the husband apparently did not influence child transmission, but most of the studied women were diagnosed after becoming mothers and information on the partner or ex-partner serological status was unknown for 40% of them. Thus, this possibility could not be totally ruled out in this study. In turn, the higher number of children per mother, which could be related to age, did not influence the transmission rate.

Mother’s schooling, as a prediction for mother income, tended to correlate inversely with the risk of seropositivity of the child, but probably the sample size was not large enough to identify this possible protecting factor. In fact, several studies reported that indicators of lower socioeconomic status, such as lower educational level and low income, are associated with HTLV-1 transmission^2^ and to MTCT^2,34,35^. Transmission occurred regardless of the sex of the child, but this data is controversial, since some studies have associated a higher risk of transmission to males^23^ or females^22^ exposed children, whereas others found a similar transmission risk for boys and girls^24,27,28,30,36,37^. However, the small sample size is a limitation to influence the child infection from other factors, such as history of blood transfusion, cesarean delivery or maternal coinfection with HIV, HTLV-2, HBV or an association of HIV/HCV.

The main limitation of this study was that most of the participants were 15 years old or older when tested. Therefore, we cannot assure that all our patients were infected through the vertical route. However, it is unlikely that all patients were infected by the sexual route at such young age. This possibility may not invalidate the major findings of our study. On the other hand, we cannot prospectively test such hypothesis due to obvious ethical issues.

It is important to highlight some independent risk factors identified in this cohort, such as a higher maternal proviral load (>100 copies/10^4^ PBMC), prolonged breastfeeding, and familial aggregation with more than one infected child in the same offspring. In fact, there is a genetic susceptibility for HTLV-1 infection^38^, but this possibility must be addressed further in another study. Thus, PVL was a predictive marker for a positive child, even when performed several years after the putative transmission from the mother. Finally, screening for HTLV-1 among pregnant women and a non-breastfeeding policy by infected mothers continue to be the main measures to prevent MTCT where this infection is endemic.
